# An Epistemological Concern

**DOI:** 10.1111/aas.70143

**Published:** 2025-10-29

**Authors:** Søren Søndergaard

**Affiliations:** ^1^ Centre of Elective Surgery, Regional Hospital of Silkeborg & Institute of Clinical Medicine University of Aarhus Aarhus Denmark

In a recent issue of *Acta Anaesthesiologica Scandinavica*, Ter Horst et al. reported a study suggesting the inclusion of photoplethysmography (PPG) in the clinical decision support armamentarium for identifying patients with sepsis who may require a vasopressor [1]. Ten features were identified in the PPG signal. These were related, inter alia, to stroke volume, vascular tone, compliance, systemic vascular resistance, and heart rate. Data were subjected to Principal Component Analysis (PCA) to extract orthogonal components that summarize their variability. Three components explained 80.3% of the variance: PC1 (compliance), PC2 (CO, SVR), and PC3 (vasomotor tone). The features were combined with vital and paraclinical parameters, sickness scorings, and hemodynamics within each component and subjected to K‐means clustering to identify subphenotypes with distinct PPG patterns. Three types emerged: Type A (lowest peripheral resistance, compliance of elastic arteries, SVR, and arterial distensibility), Type B (highest peripheral resistance and arterial compliance), and Type C (low CO, SVR, and peripheral resistance). Finally, PC1–PC3, along with confounding demographic and comorbidity data, were entered into a multivariable logistic regression. PPG features alone had a moderate ability to identify patients receiving vasopressors in the ICU, with an AUROC of 0.746, a sensitivity of 69%, a specificity of 76%, and positive and negative predictive values of 94% and 32%, respectively.

The study represents a welcome and stimulating exploration in the field of “hemodynamic reflections”, that is, measurable changes in variables resulting from the circulation. Most prominent in this province of intensive care are the dynamic indices, where the external excitation of the cardiovascular system elicits changes in stroke volume, pulse pressure, blood pressure, capillary refill time, or heart rate (AI enumerates 18 variables derived from dynamic preload, and autonomic/vascular reactivity tests, electrophysiology, and perfusion markers). Excitation may present as an abrupt change in PEEP level, tidal volume, respiratory frequency, or a change in position, and is used as a form of leverage. PPG, on the other hand, registers a variation inherent to the plethysmogram without external excitation. It has no generic term (like ‘dynamic indices’), but may suitably be referred to as an ‘inherent index’. Both types of indices are “one step removed” from the actual variable (CO, BP, HR) and are thus influenced by cardiovascular and pulmonary physiology. For example, as indices of fluid responsiveness, the ΔETCO_2_ is dependent on $\dot{V}{\mathrm{CO}}_{2}$, pulmonary blood flow and V_A_, and ΔSV is influenced by the autonomic homeostatic reflexes during passive leg raising. Likewise, the pulse pressure and stroke volume variations will be affected by reduced venous return if supranormal tidal volumes are used. So, one may ask, do the circuitous use of inherent and dynamic indices when obtaining information about the circulation actually perform the task without numerous exceptions and limitations? In the following paragraphs, further questions are raised for the contemplation of the authors (and others), so they may safely, if possible, reach their vision: “… future work should focus on improving the technique and methodology, performing robust external validation, and developing practical real‐time bedside tools with actionable thresholds for integration into sepsis resuscitation protocols.”

Inherent and dynamic indices aside, the study, furthermore, raises questions about the choice of the cardiovascular model and statistical analyses. These may be subsumed under the heading of ‘the epistemological concern’.

Focusing on the model, it should describe the physiological and pathophysiological foundations, describe the key elements, explain how they interact, and predict outcomes based on treatments. To achieve this, the model should be both explanatory and predictive, as well as pragmatic and consistent in its application of causality. Some add ‘beautiful’ and adduce the Hodgkin‐Huxley model describing the ionic basis of the action potential in neurons [2]. Ultimately, the cardiovascular model must show measurable benefits in reducing morbidity and mortality [3, 4]. Notably, the model using PLR has yielded only minimal improvements in morbidity and mortality [5]. Popper held that a model can be either supported, that is, never proven to be true, or falsified by testing its explanatory power and predictive accuracy. If falsified, it must be reformulated to provide an improved explanation and prediction [6]. The MODEL is integral to the four‐leafed 4 M concept of MISSION (the aim of the measurement or intervention), MEANS (the means for executing the measurement or intervention), and MONITORING (the surveillance of obtaining the measurement or completion of the mission using the chosen means) [7]. This PPG study lacks a disease model of septic shock in pathophysiological, infectious, and immunological terms and a cardiovascular model explaining how the features—SPA, DPA, CT, DT, IPA, PI, PW, RI, APG, and PPI—relate to a cardiovascular model of primary determinants and derived variables, and how the disease model interacts with the cardiovascular model.

The choice of statistics amounts to answering the question of which analyses will examine the model's fit to the data, making scientific sense when interpreting the parameter values [8].

Without a model, the authors attempt to bring order to a seemingly chaotic collection by applying Principal Component Analysis (PCA) to organize the data [9]. PCA has been applied to various fields, including imaging, EEG, ECG, gene expression studies, biomarker response, and symptom clustering. PCA has demonstrated the capacity for dimensionality reduction, noise reduction, feature extraction, exploratory data analysis, and handling collinearity in regression. The analytic steps in PCA are transparent to statisticians and enigmatic to non‐statisticians.

1. Standardize the dataset.
2. Calculate the covariance matrix for the features in the dataset.
3. Calculate the eigenvalues and eigenvectors for the covariance matrix.
4. Sort eigenvalues and their corresponding eigenvectors.
5. Pick k eigenvalues and form a matrix of eigenvectors.
6. Transform the original matrix.

Three caveats warn against PCA; several of these appear relevant here:

When mechanistic interpretability is needed: PCA may obscure meaning.

When variance ≠ importance: PCA assumes directions of maximum variance are the most important; clinically relevant changes may occur in low‐variance directions.

When data are non‐linear: physiological systems are often non‐linear; when biological prior knowledge exists: PCA may obscure rather than clarify the physiology.

By combining PPG analysis with PCA, the study appears to have sidestepped the arduous task of irreducible complexity in building a model, providing a mission, means, and monitoring, offering a causal relation between the signal changes in the 10 POIs and the hemodynamic events, and choosing the statistical techniques that will fit the model to the data. In a similar vein, the PPVs and NPVs of multivariable logistic regression may create ambiguity. They do not predict the future; they are conditional probabilities based on existing data. “Predictive” means predictive of truth (disease status), not predictive of future outcomes [8]. The authors are not alone in this misconception: the ‘prediction’ deduced from ROC analyses in dynamic indices (PPV, SVV, PLR, et sim.) is merely ‘postdiction’ with restricted bearing on future cases. It may be predicted that the realization of this fact has dire implications for the foundations of Goal‐Directed Therapy and Functional Hemodynamic Therapy. Deprived of 4 M, the present analyses remain a preliminary and uncertain estimate. The Anon quote may now be updated to “Give a man four weapons—correlation, regression, a pen, and PCA—and he will use all four” [10].

This type of exploratory observational research, which does not employ explicit disease or cardiovascular models, highlights both the promise and the limitations of inductive methods in advancing our understanding of critical illness. While such approaches can identify associations and patterns that inform hypothesis generation, they also present challenges for drawing robust causal inferences, especially in the absence of clear mechanistic models. These constraints are well recognized within the broader field of intensive care research, where definitive causality is inherently complex to establish.

At the same time, the findings of this study appear within established, validated models and prior clinical experience, aligning with the body of evidence characterizing circulatory events and physiological adaptations in critically ill patients. This further underscores the complexity and multifactorial nature of cardiovascular phenomena in intensive care settings and where to look for answers.

Looking ahead, future studies in this area may benefit from integrating explicit mechanistic or computational models alongside observational data, thus advancing the field toward greater causal clarity. The ongoing development and testing of such models, as well as more precise hypothesis formulation and experimental validation, could help bridge the gap between descriptive analytics and actionable understanding. Ultimately, the refinement of research methods and the cumulative integration of diverse approaches will be essential for unraveling the intricacies of circulatory pathophysiology and improving patient outcomes in critical care [11].

## Author Contributions

Sø conceived the editorial, wrote and accepted the draft.

## Conflicts of Interest

The author declares no conflicts of interest.

## Data Availability

The author has nothing to report.
